# Combined Effects of PPAR**γ** Agonists and Epidermal Growth Factor Receptor Inhibitors in Human Proximal Tubule Cells

**DOI:** 10.1155/2013/982462

**Published:** 2013-02-24

**Authors:** Katherine Pegg, Jie Zhang, Carol Pollock, Sonia Saad

**Affiliations:** Department of Medicine, Kolling Institute of Medical Research, Northern Clinical School, University of Sydney, Australia

## Abstract

We aimed to determine whether epidermal growth factor receptor (EGFR) inhibition, in addition to a peroxisome proliferator-activated receptor gamma (PPAR**γ**) agonist, prevents high-glucose-induced proximal tubular fibrosis, inflammation, and sodium and water retention in human proximal tubule cells exposed to normal glucose; high glucose; high glucose with the PPAR**γ** agonist pioglitazone or with the P-EGFR inhibitor, gefitinib; or high glucose with both pioglitazone and gefitinib. We have shown that high glucose increases AP-1 and NF**κ**B binding activity, downstream phosphorylation of EGFR and Erk1/2, and fibronectin and collagen IV expression. Pioglitazone reversed these effects but upregulated NHE3 and AQP1 expression. Gefitinib inhibited high glucose induced fibronectin and collagen IV, and EGFR and Erk1/2 phosphorylation and reversed pioglitazone-induced increases in NHE3 and AQP1 expression. Our data suggests that combination of an EGFR inhibitor and a PPAR**γ** agonist mitigates high-glucose-induced fibrosis and inflammation and reverses the upregulation of transporters and channels involved in sodium and water retention in human proximal tubule cells. Hence EGFR blockade may hold promise, not only in limiting tubulointerstitial pathology in diabetic nephropathy, but also in limiting the sodium and water retention observed in patients with diabetes and exacerbated by PPAR**γ** agonists.

## 1. Introduction

Cellular sodium and water transport are dysregulated in diabetes mellitus resulting in volume-mediated hypertension and cardiac complications. We have previously demonstrated that epidermal growth factor (EGF) and high-glucose-induced sodium reabsorption in proximal tubule cells by increasing the activity of the sodium hydrogen exchanger-3 (NHE3). This is dependent on EGFR signalling and downstream activation of serum and glucocorticoid-inducible kinase (Sgk-1) [1]. Enhanced expression and/or activity of the EGF receptor (EGFR) has previously been observed in the kidneys of diabetic animals [2] and tubular EGFR expression correlates with the extent of interstitial fibrosis [3]. Furthermore, the EGFR is activated/transactivated by multiple factors inherent in the diabetic milieu, including high glucose [4], angiotensin II (AngII) [5], and aldosterone [6], all of which have been implicated in the pathogenesis of diabetic nephropathy. Recent studies have supported the hypothesis that inhibition of the EGFR provides an attractive therapeutic target for the treatment of diabetic nephropathy [7].

Thiazolidinediones (TZDs) are synthetic peroxisome proliferator-activated receptor gamma (PPAR*γ*) agonists, currently used in the treatment of type 2 diabetes as hypoglycaemic agents. TZDs have been shown to reduce albuminuria and decrease glomerular matrix deposition, glomerulosclerosis, and tubulointerstitial fibrosis in insulin deficient and insulin resistant diabetic rat models [8, 9], and in humans with diabetic nephropathy [10, 11]. Hence they have properties that suggest superior benefit in patients with diabetes mellitus at risk of nephropathy. However, their use is limited by fluid retention especially in those with concomitant overt or incipient cardiac failure [12], which may contribute to the excess cardiovascular event rate observed in patients treated with specific PPAR*γ* agonists [13]. The mechanisms by which PPAR*γ* agonists upregulate sodium and water transport in the human kidney are via enhanced NHE3 activity in proximal tubule cells [14], or through the distal tubular epithelial sodium channel [15]. Concurrent increases in water transport occur via enhanced flux primarily through aquaporin-1 (AQP1) in proximal tubule cells. Our recent research suggests signalling via Sgk-1 may represent a common mechanism whereby both salt and water transport are enhanced by PPAR*γ* agonists [15] and conversely may be limited by EGFR antagonists.

Hence we hypothesise that the EGFR plays a role in the development of diabetic nephropathy as well as in the associated sodium and water retention, which is exacerbated by concomitant treatment with PPAR*γ* agonists. The role of the EGFR in PPAR*γ* agonist mediated sodium retention and the combined effects of PPAR*γ* agonists and EGFR inhibitors in an *in vitro *model of human tubular cells were tested in this study.

## 2. Materials and Methods

### 2.1. Cell Culture

**Figure 1:**
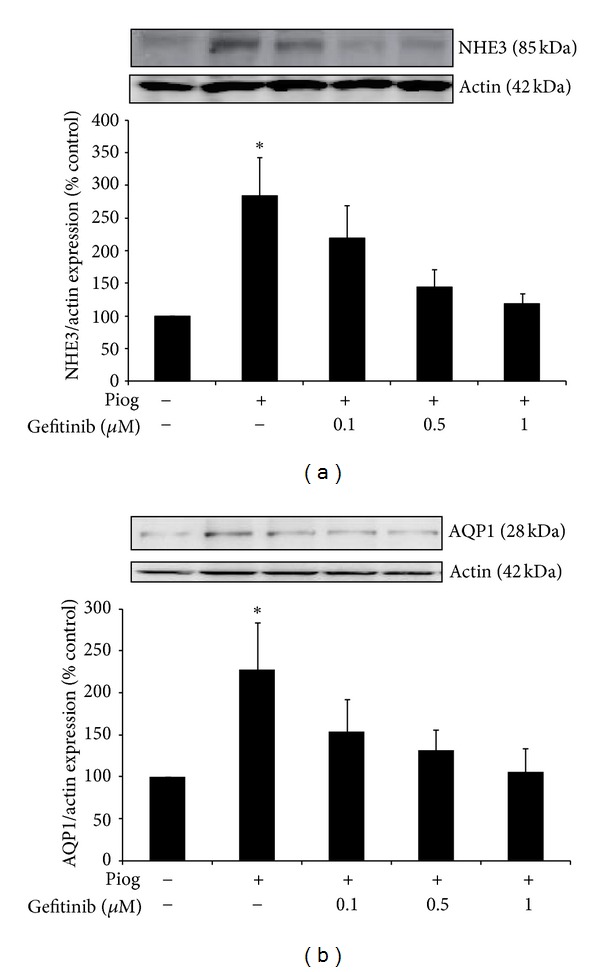
Gefitinib dose response in human HK2 cells showing inhibition of pioglitazone- (Piog-) induced NHE3 (a) and AQP1 (b) in the presence of 0.1–1 *μ*M of gefitinib. HK2 cells were studied for 48 hrs. Western blot was determined as described in Section 2. Representative images for NHE3, AQP1, and actin bands are shown. Normalized results are expressed as mean ± SEM; *n* = 3. *P < 0.05 versus control.

Human kidney-2 (HK-2) cells, an immortalized human kidney proximal tubule cell line from American Type Cell Collection (ATCC, USA), were used in this study. Cells were grown in keratinocyte serum-free media (Invitrogen, USA) and seeded at 80–90% confluence prior to exposure to the following experimental conditions for 48 hrs: 5 mM d-glucose (normal glucose) and 30 mM d-glucose (high glucose) ±10 *μ*M pioglitazone. To determine the role of the EGFR in mediating observed changes, the EGFR tyrosine kinase inhibitor gefitinib was used in cells exposed to either high glucose or a combination of both high glucose and pioglitazone. Initial “dose-response” experiments were undertaken to determine the concentration at which pioglitazone maximally stimulated PPAR-*γ* protein expression. Based on these studies, 10 *μ*M of pioglitazone was used in the experimental protocols as previously described [14, 16, 17]. A dose response study was performed to determine the optimal concentration of gefitinib needed to block the EGFR phosphorylation and its downstream signalling pathway (Figure 1). Pioglitazone was purchased from Alexis Chemicals, USA, and gefitinib from AstraZeneca, UK.

### 2.2. Western Blotting

Western blots were performed on Triton X-100 soluble fractions. AQP1 and NHE3 antibodies (Chemicon International), Fibronectin (Sigma Aldrich), Collagen IV (Abcam), P-EGFR antibody (pY1068, Invitrogen), total EGFR antibody, P-Erk1/2 and total Erk1/2 (cell signaling), or actin antibody (Sigma) were used overnight followed by incubation with antirabbit or antimouse antibody (Amersham Pharmaceuticals) for 1 hr at room temperature. The bands corresponding to AQP-1 (28 KDa), NHE3 (85 KDa), P-EGFR (170 KDa), t-EGFR (175 KDa), P-Erk1/2, t-Erk1/2 (44 and 42 KDa), fibronectin (220 KDa), collagen IV (200 KDa), and actin (42 KDa) were quantified using NIH Image soft v1.60.

### 2.3. Electrophoretic Mobility Shift Assay (EMSA)

After exposure to the above-mentioned experimental conditions, nuclear extract was prepared using NucBuster Protein Extraction Kit (Novagen) according to the manufacturer's instructions. A digoxygenin (DIG) Gel Shift Kit (Roche Applied Science, Indianapolis, IN) was used in the EMSA. In brief, 25 *μ*g of nuclear extract was incubated with 1 *μ*g poly [d(I-C)] as the nonspecific competitor, 1 *μ*g poly L-lysine in a binding buffer [(in mM) 100 HEPES, pH 7.6, 5 EDTA, 50 (NH_4_)_2_SO_4_, 5 DTT, and 150 KCl, as well as 1% Tween 20, wt/vol], and DIG-labeled AP-1 (5′-CGC TTG ATG AGT CAG CCG GAA-3′) or NF-*κ*B (5′-AGT TGA GGG GAC TTT CCC AGG C-3′) consensus oligonucleotide (Promega) for 30 min at room temperature. Unlabeled AP-1 and NF-*κ*B consensus oligonucleotides were used as specific competitors, respectively. The reaction mixture was electrophoresed through 6% polyacrylamide gels, transferred onto positively charged nylon membrane (Roche Applied Science), and then cross-linked using an UV transilluminator for 3 min. The membrane was subjected to immunological detection using anti-DIG-AP conjugate and chemiluminescence. Shift bands were measured and analysed by Image J software and results were expressed as a percentage of control values.

### 2.4. Statistical Analysis

All *in vitro* results are expressed as a percentage of the control value. Experiments were performed in at least three different culture preparations, and at least three data points for each experimental condition were measured in each preparation. Results are expressed as mean ± SEM, with *n* reflecting the number of culture preparations. Statistical comparisons between groups were made by ANOVA with pairwise multiple comparisons made using unpaired *t*-test. Analyses were performed using the software package Statview v4.5 (Abacus Concepts, CA, USA); values ≤ 0.05 were considered significant.

## 3. Results 

### 3.1. Pioglitazone-Induced NHE3 and AQP1 Is P-EGFR Dependent

We have previously demonstrated that pioglitazone induces NHE3 and AQP1 protein expression in human primary proximal tubule cells [14]. In order to determine if EGFR is involved, experiments were repeated in the presence of the EGFR tyrosine kinase selective inhibitor, gefitinib. We have confirmed that pioglitazone induces NHE3 and AQP1 in human HK2 cells to 284 ± 59% and 228 ± 97%, respectively (both *P* < 0.05 (Figures 1(a) and 1(b)). Increasing concentration of gefitinib (0.1 to 1 *μ*M) inhibited pioglitazone-induced NHE3 in a dose dependent manner. NHE3 expression was reduced to 219 ± 50%, 144 ± 18%, and 119 ± 15%, respectively (Figure 1(a)) and AQP1 expression to 154 ± 38%, 141 ± 25%, and 97 ± 29%, respectively (Figure 1(b)).

### 3.2. Gefitinib Reduces EGFR Phosphorylation and Its Downstream Signalling

**Figure 2:**
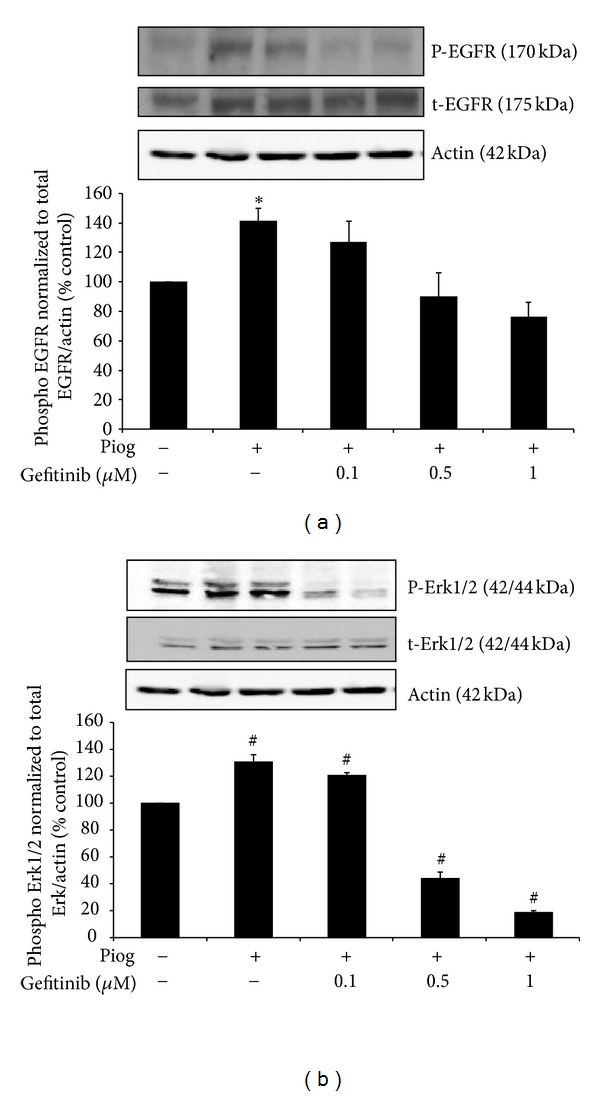
Gefitinib dose response in human HK2 cells showing a complete inhibition of high-glucose- (HG-) induced P-EGFR over t-EGFR normalized to Actin (a) and P-ErK1/2 over t-ErK1/2 normalized to Actin (b) in the presence of 0.1–1 *μ*M of gefitinib. HK2 cells were treated for 48 hrs. Western blot was determined as described in Section 2. Representative images are shown. Normalized results are expressed as mean ± SEM; *n* = 4. *P < 0.05 and ^#^ *P* < 0.0001 versus control.

We have previously demonstrated that high glucose increases P-EGFR expression in human proximal tubule cells [1]. We have confirmed that pioglitazone increases P-EGFR in human HK2 cells to 142 ± 9%  *P* < 0.05 (Figure 2(a)). Using different concentration of gefitinib (0.1–1 *μ*M), we have clearly demonstrated that pioglitazone induced P-EGFR expression is reduced in a dose dependent manner to 127 ± 14%, 90 ± 16%, and 76 ± 10%, respectively (Figure 2(a)). Pioglitazone also increases P-Erk1/2 expression to 131 ± 5%,  *P* < 0.0001 (Figure 2(b)) and P-Erk1/2 was similarly reduced by increasing doses of gefitinib (0.1–1 *μ*M) to 121 ± 2%, 44 ± 5%, and 19 ± 2% (*P* < 0.0001), respectively (Figure 2(b)). Based on these experiments, 0.5 *μ*M was used for the remainder of the experiments. No cell toxicity, as determined by the MTT proliferation assay, was demonstrated using this concentration (data not shown).

### 3.3. Gefitinib Reduces High-Glucose-Induced AQP1 in the Presence and Absence of Pioglitazone

**Figure 3:**
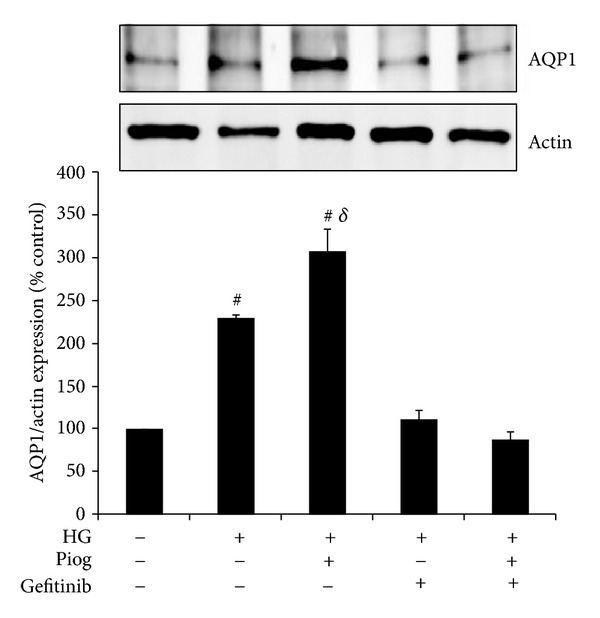
High glucose and pioglitazone induced AQP1 are mediated through EGFR phosphorylation. HK2 cells were incubated for 48 h with 5 mM glucose media (control), high glucose (HG) ± Piog (10 *μ*M) ± gefitinib (0.5 *μ*M) and levels of AQP1 were determined by Western blotting. Representative images for AQP1 and actin bands are shown. Normalized results are expressed as mean ± SEM; *n* = 3. ^#^ *P* < 0.0001 versus control and ^*δ*^ *P* < 0.005 versus HG.

As expected, our data demonstrate that high glucose increases AQP1 protein expression to 230 ± 4% of control values (*P* < 0.0001). This is further increased in the presence of pioglitazone to 308 ± 26% of control values (*P* < 0.0001). Importantly, the use of gefitinib completely inhibited high glucose and pioglitazone induced AQP1 expression to 111 ± 11% and 87 ± 10% of control values, respectively (Figure 3). These results suggested that high glucose and pioglitazone-increased AQP1 expression are mediated though EGFR phosphorylation.

### 3.4. Gefitinib Reduces High-Glucose-Induced NHE3 in the Presence and Absence of Pioglitazone

**Figure 4:**
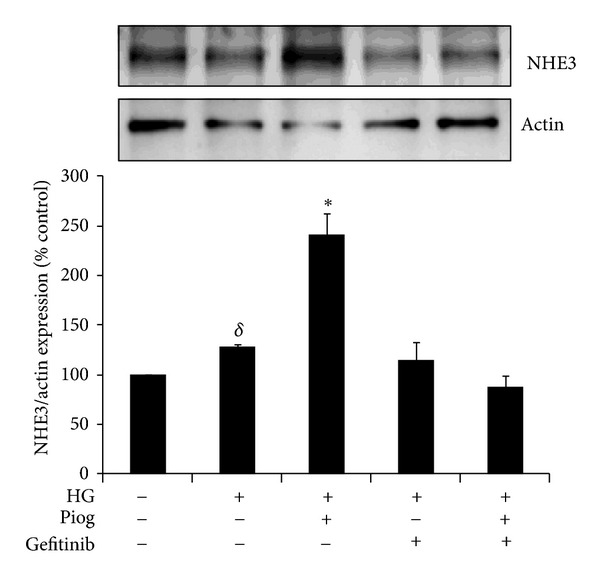
High glucose and pioglitazone induced NHE3 expression are mediated through EGFR phosphorylation. HK2 cells were incubated for 48 h with 5 mM glucose media (Control), high glucose (HG) ± Piog (10 *μ*M) ± gefitinib (0.5**** *μ*M) and levels of NHE3 were determined by Western blotting. Representative images for NHE3 and actin bands are shown. Normalized results are expressed as mean ± SEM; *n* = 3. *P < 0.05 versus Control and ^*δ*^ *P* < 0.005 versus Control.

We have previously demonstrated that high glucose and pioglitazone independently increase NHE3 expression in human proximal tubule cells [1, 14]. Our data confirms that high glucose increased NHE3 in HK2 cells to 118%  ±3% versus control (*P* < 0.005). Pioglitazone significantly increases the high glucose induction of NHE3 to 158 ± 22% versus control (*P* < 0.05). Gefitinib reversed NHE3 expression with high glucose and pioglitazone to baseline levels 95 ± 17% and 79 ± 11%, respectively (Figure 4). These results clearly demonstrate that the high glucose and pioglitazone induced increases in NHE3 are mediated though EGFR phosphorylation. 

### 3.5. Pioglitazone and Gefitinib Reduce High-Glucose-Induced Fibronectin and Collagen IV Expression

**Figure 5:**
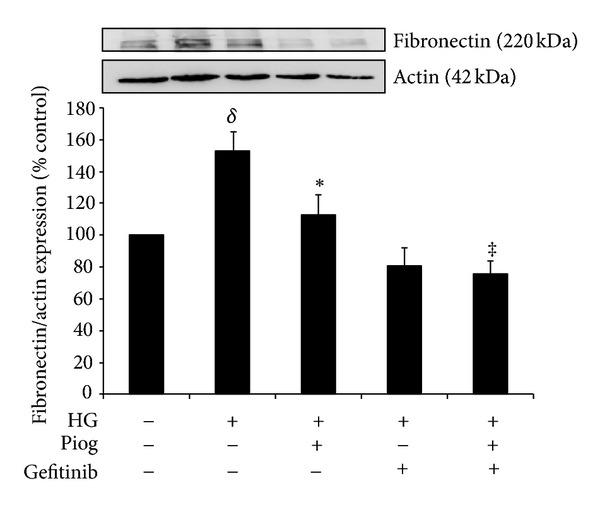
High-glucose-induced fibronectin expression. Pioglitazone and gefitinib reduced high-glucose-induced fibronectin expression. Combination of both pioglitazone and gefitinib has a similar effect. HK2 cells were incubated for 48 h with 5 mM glucose media (control), high glucose (HG) ± Piog (10 *μ*M) ± gefitinib (0.5**** *μ*M) and levels of fibronectin were determined by Western blotting. Representative images for fibronectin and actin bands are shown. Normalized results are expressed as mean ± SEM; *n* = 4. ^*δ*^ *P* < 0.005 versus control and *P < 0.05 versus HG and ^‡^ *P* < 0.05 versus HG + Piog.

**Figure 6:**
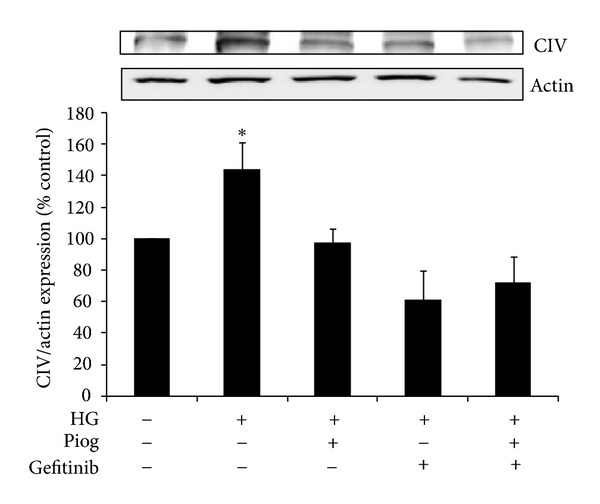
High-glucose-induced collagen IV (CIV) expression. Pioglitazone and gefitinib reduced high-glucose-induced CIV expression. The combination of both, pioglitazone and gefitinib has a similar effect. HK2 cells were incubated for 48 h with 5 mM glucose media (control), high glucose (HG) ± Piog (10**** *μ*M) ± gefitinib (0.5**** *μ*M) and levels of CIV were determined by Western blotting. Representative images for CIV and actin bands are shown. Normalized results are expressed as mean ± SEM; *n* = 3. *P < 0.05 versus control.

Our data demonstrates that high glucose increases fibronectin and collagen IV expression to (153 ± 11% and 144 ± 17%, resp.; *P* < 0.05) (Figures 5 and 6). As expected, and in keeping with the known protective effect of pioglitazone on fibrosis, we have demonstrated that pioglitazone reduces high glucose increased fibronectin and collagen IV protein expression to (113 ± 12% and 97 ± 9%, resp.). In order to determine whether inhibiting EGFR phosphorylation has any beneficial effect on fibrosis or whether it affects the antifibrotic effects of PPAR*γ* agonist when used in combination, gefitinib effect on fibronectin and collagen IV was determined in the presence and absence of pioglitazone. Our data demonstrate that gefitinib reduced high glucose increased fibronectin expression to 81 ± 11% and potentiated pioglitazone's effects to (76 ± 8%, *P* < 0.05 versus high glucose + pioglitazone) (Figure 5). Gefitinib similarly reduced high glucose increased collagen IV expression to 61 ± 18%. Gefitinib had no additional effects on collagen IV expression when compared to pioglitazone (72 ± 16%) (Figure 6).

### 3.6. Pioglitazone and Gefitinib Reduce Increases in AP-1 Binding Activity Induced by High Glucose

**Figure 7:**
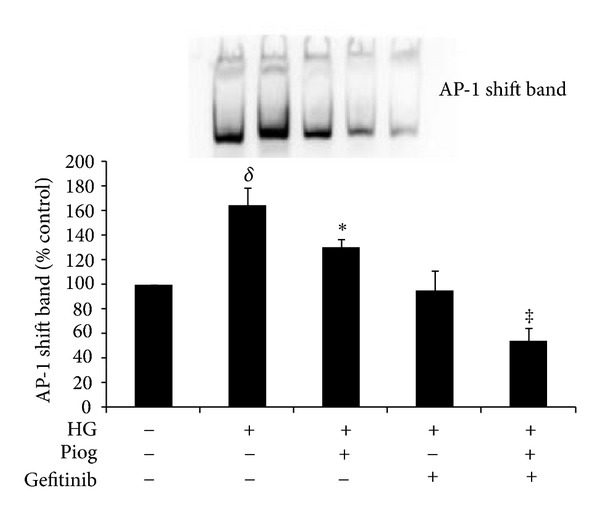
High-glucose-induced DNA binding of DIG-labelled AP-1. AP-1-DNA binding activity was significantly increased with high glucose. Pioglitazone reduces high-glucose-induced AP-1 binding activity. Gefitinib completely abolished AP-1 binding activity induced by HG. HK2 cells were incubated for 72 hrs with 5 mM glucose media (Control), high glucose (HG) ± Piog (10**** *μ*M) ± gefitinib (0.5**** *μ*M). Nuclear extract preparation and EMSA are as described in Section 2. A representative image showing the shift band of AP-1 is shown. Quantitative data are expressed as mean ± SEM; *n* = 3. ^*δ*^ *P* < 0.005 versus control; *P < 0.05 versus Control and ^‡^ *P* < 0.001 versus HG + Piog.

As expected, high glucose increased the DNA binding of DIG-labelled AP-1 to 164 ± 14% of control (*P* < 0.005). This was reduced significantly in the presence of pioglitazone to 130 ± 7%; *P* < 0.05). Furthermore,, gefitinib reduced high glucose increased binding of DIG-labelled AP-1 to 95 ± 16%; (*P* < 0.005 versus high glucose) and further potentiated the effect of pioglitazone on AP-1 binding activity to 54 ± 11%; (*P* < 0.001 versus high glucose and pioglitazone; Figure 7).

### 3.7. Pioglitazone and Gefitinib Reduce NF*κ*B Binding Activity Induced by High Glucose

**Figure 8:**
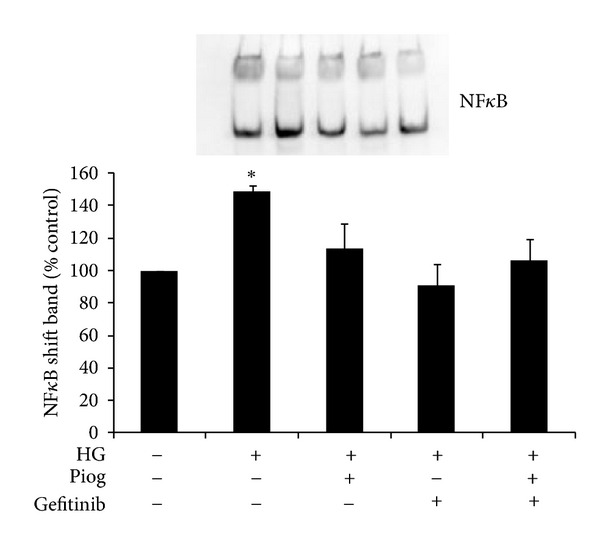
High-glucose-induced DNA binding of DIG-labeled NF*κ*B. NF*κ*B-DNA binding activity was significantly increased with high glucose. Pioglitazone significantly reduces high-glucose-induced NF*κ*B binding activity. Gefitinib has a similar effect. HK2 cells were incubated for 72 hrs with 5 mM glucose media (Control), high glucose (HG) ± Piog (10**** *μ*M) ± gefitinib (0.5**** *μ*M). Nuclear extract preparation and EMSA are as described in Section 2. A representative image showing the shift band of NF*κ*B is shown. Quantitative data are expressed as mean ± SEM; *n* = 3. *P < 0.05 versus control.

Our data demonstrate that high glucose increases the DNA binding of DIG-labelled NF*κ*B to 146 ± 4% of control (*P* < 0.05). This was reduced to basal levels in the presence of pioglitazone to 105 ± 15%; (*P* < 0.05 versus high glucose). Gefitinib reduced the high glucose and pioglitazone induced increased binding of DIG-labelled NF*κ*B to 84 ± 13% and 101 ± 13%, respectively; *P* < 0.005 and *P* < 0.05 (Figure 8). 

## 4. Discussion

Extensive literature exists on the beneficial effects of synthetic PPAR*γ* agonists, and in particular pioglitazone, in limiting insulin resistance, protecting pancreatic *β* cell function and our own data suggest a protective effect on nephropathy [16–20]. Despite an observed increase in cardiovascular event rates, novel PPAR*γ* agonists are still in development [13], largely because of their efficacy and potential benefits in addition to improving glycaemic control. It is recognised that the increase in cardiovascular event rates in patients with impaired renal function are at least in part due to volume overload [21]. Salt and water retention are key side effects limiting the use of PPAR*γ* agonists in clinical practice, potentially contributing to the increased incidence of cardiovascular events in patients utilising these drugs. Hence determining the cause of the sodium and water retention induced by PPAR*γ* agonists and developing strategies to limit its occurrence are of key importance.

The underlying mechanisms of TZD-induced plasma volume expansion and oedema are potentially manifold. We have previously demonstrated that high glucose and the PPAR*γ* ligand, pioglitazone, induce PPAR*γ* expression [16] and that PPAR*γ* agonists upregulate the main sodium and water channels in the proximal tubule, NHE3 and AQP1 protein expression, through an Sgk1 mediated pathway, which is considered to be a significant modifier of cellular volume in conditions of osmotic stress [22]. We have demonstrated that high glucose and pioglitazone induced NHE3 and AQP1 are completely inhibited by gefitinib, the clinically available tyrosine kinase inhibitor. This suggests that inhibiting EGFR activation may be beneficial, not only in preventing sodium and water retention generally observed in patients with diabetic nephropathy, but also in limiting sodium and water retention associated with the use of PPAR*γ* agonists in patients with diabetes mellitus. To date, few studies have focused on the role of the EGFR in fluid retention in disease states. It is known that EGF enhances solute and water reabsorption in the kidney proximal convoluted tubule [23]. It has been more recently reported that EGFR activation may contribute to the development of hypertension by regulating vascular tone and renal sodium handling [21]. Hence EGFR inhibitors have been used to reduce blood pressure in experimental models of hypertension, suggesting that EGFR is a novel target for salt sensitive hypertension [24]. We have previously demonstrated that EGF and high-glucose-induced NHE3 expression and activity in the proximal tubule is EGFR dependent with downstream activation of Sgk1 [1]. Furthermore, we and others have demonstrated that angiotensin II and aldosterone upregulate NHE3 expression and activity in the proximal tubule via EGFR-dependent mechanism [25, 26]. These data suggest a central role for the EGFR in sodium and water homeostasis, which is clearly a key factor in extracellular volume and blood pressure regulation. The effect of EGFR tyrosine kinase inhibition in the regulation of sodium transport in the proximal tubule opens an attractive area for potential therapeutic intervention. 

EGFR signalling is a key initiator of multiple signalling pathways in the development of diabetes-induced vascular dysfunction [27] and renal pathology. EGFR is upregulated in various forms of renal disease including diabetic nephropathy, glomerulonephritis, and allograft nephropathy [3, 28–31]. Tubular EGFR expression correlates with the extent of interstitial fibrosis [3]. We and others have previously demonstrated that EGFR is upregulated in the kidneys of animals with experimentally induced diabetes [32] and high glucose regulates its transactivation [1, 4]. Our present data uniquely demonstrate that high-glucose-induced fibronectin and collagen expression in the proximal tubule are dependent on EGFR phosphorylation. 

We have clearly shown for the first time that gefitinib reduces high glucose mediated fibronectin, collagen IV expression, and high-glucose-induced increases in activity of both NF*κ*B and AP-1. Importantly, Gefitinib potentiated the beneficial effect of pioglitazone in reducing fibronectin expression and AP-1 promoter activity. Since the fibronectin promoter contains an AP-1-binding site [33], it is highly likely that the high glucose and pioglitazone effect on fibronectin expression is through the regulation of AP-1 promoter activity, which is potentiated by EGFR phosphorylation. The ability of gefitinib to inhibit NF*κ*B and AP-1 activity suggests a beneficial role for targeting EGFR in diabetic nephropathy, especially since NF*κ*B and AP-1 binds to the promoter regions of, and hence is able to regulate, several genes thought to be important in the pathogenesis of nephropathy, including transforming growth factor-*β*1 (TGF*β*1) and monocyte chemotactic protein-1 (MCP-1) [34–36]. We have previously demonstrated that a reduction in tubular production of MCP-1 is associated with an upregulation of PPAR*γ* [16]. Our results suggest that the AP-1 pathway, modified by PPAR*γ* agonist activity and EGFR blockade, is likely to be responsible for reduction of several factors known to be involved in profibrotic and also proinflammatory pathways.

Liu et al. has recently demonstrated that EGFR may mediate renal fibrogenesis by promoting transition of renal epithelial cells to a profibrotic phenotype, increased production of inflammatory factors, and activation of renal interstitial fibroblasts [37]. Diabetic rats that received an EGFR tyrosine kinase inhibitor show attenuated kidney and glomerular enlargement and reduction in albuminuria in association with podocyte preservation [38, 39]. EGFRs have been recognised as key targets in anticancer therapy and EGFR inhibitors are increasingly used in the treatment of cancer with no reported renal toxicity, despite such patients being at increased risk of acute renal injury, predominantly due to sepsis. The major side effect of targeting the EGFR clinically is inflammatory reactions in the skin [24]. Hence targeting the EGFR in combination with the PPAR*γ* agonist has the potential to be beneficial in regulating nephromegaly, matrix expansion, and fibrosis in addition to excessive sodium reabsorption observed in diabetic nephropathy.

## 5. Conclusions

In summary, we have demonstrated that EGFR inhibitors prevent upregulation of pathways that are implicated in the sodium and water retention seen in patients with diabetes mellitus and exacerbated by the use of PPAR*γ* agonists. Furthermore, they have a synergistic effect on limiting high-glucose-induced upregulation of inflammatory and fibrotic pathways when used with PPAR*γ* agonists, which is likely to ameliorate the tubulointerstitial pathology observed in diabetic nephropathy.

## Abbreviations

P-EGFR: Phospho-epidermal growth factor receptor
NHE3: Sodium-hydrogen exchanger-3
AQP-1: Aquaporin-1.
